# Validating large language models against manual information extraction from case reports of drug-induced parkinsonism in patients with schizophrenia spectrum and mood disorders: a proof of concept study

**DOI:** 10.1038/s41537-025-00601-5

**Published:** 2025-03-20

**Authors:** Sebastian Volkmer, Alina Glück, Andreas Meyer-Lindenberg, Emanuel Schwarz, Dusan Hirjak

**Affiliations:** 1 Department of Psychiatry and Psychotherapy, Central Institute of Mental Health, Medical Faculty Mannheim, Heidelberg University, Mannheim, Germany; 2 Hector Institute for Artificial Intelligence in Psychiatry, Central Institute of Mental Health, Medical Faculty Mannheim, Heidelberg University, Mannheim, Germany; 3 German Centre for Mental Health (DZPG), Partner Site Mannheim-Heidelberg-Ulm, Mannheim, Germany

**Keywords:** Biomarkers, Neuroscience

## Abstract

In this proof of concept study, we demonstrated how Large Language Models (LLMs) can automate the conversion of unstructured case reports into clinical ratings. By leveraging instructions from a standardized clinical rating scale and evaluating the LLM’s confidence in its outputs, we aimed to refine prompting strategies and enhance reproducibility. Using this strategy and case reports of drug-induced Parkinsonism, we showed that LLM-extracted data closely align with clinical rater manual extraction, achieving an accuracy of 90%.

## Introduction

Innovations in natural language processing (NLP) have successfully demonstrated the utility of applying novel machine learning (ML) algorithms to mine unstructured clinical information. NLP techniques are, however, still in their conceptual stages^1,2^, and models often require additional training data for fine-tuning. Recent developments have highlighted the potential of large language models (LLMs) as an emerging technology for extracting valuable insights from unstructured clinical records through prompting strategies without explicit fine-tuning or need for additional training^3,4^. While previous NLP studies have focused on data mining electronic health records (EHR)^1^, the analysis of existing case reports is still rare, especially in the field of psychiatry. The analysis of case reports could be valuable for gaining clinical insights in underexamined psychiatric fields where high-quality randomized clinical trials, meta-analyses, and large-scale phenomenological studies are limited. This may enable the extraction of valuable information about such conditions and contribute to refining treatment guidelines in the absence of robust clinical trial and large-scale data.

This study aimed to investigate whether LLMs could leverage an established clinical rating scale and its instructions like the Unified Parkinson’s Disease Rating Scale (UPDRS) part III (motor examination), to extract information from previously published case reports on drug-induced parkinsonism (DIP)^5,6^ without prior training or fine-tuning of the model. By utilizing these preexisting instructions we aimed to standardize and improve prompting strategies for mining clinical information in psychiatry which is important for improving model performance^7^.

While DIP has been studied in psychiatry, most investigations have relied on traditional clinical methodologies rather than automated text analysis. The use of LLMs to extract relevant clinical data from case reports represents a novel strategy to systematically analyze existing literature and identify patterns that might otherwise be overlooked. DIP is the second most common cause of parkinsonian symptoms after Parkinson’s disease (PD), with prevalence estimates ranging from 20 to 35% among patients with schizophrenia spectrum disorders (SSD) who are taking antipsychotics^8^. Additionally, other medications, including tricyclic antidepressants (TCAs), selective serotonin reuptake inhibitors (SSRIs), and mood stabilizers (e.g., lithium and valproate), are known to cause DIP. Despite the recognition of DIP being exacerbated by commonly prescribed medications, debates persist regarding the prevalence rates and clinical characteristics of psychiatric patients with DIP. Therefore, examining the epidemiology, phenomenology, and treatment strategies of DIP in SSD and mood disorders (MOD) is crucial. Utilizing LLM analysis of case reports can provide valuable insights, as randomized clinical trials, meta-analyses, and large-scale studies on DIP in SSD and MOD (and other mental disorders) remain scarce.

As part of this investigation, we employed GPT-4o to analyze identified DIP case reports and series, using standardized prompts derived from psychiatric rating scales. Instructing GPT-4o to assess its confidence levels, we explored whether this could improve alignment between the LLM’s responses and medical expert assessments, particularly in light of the potential computational demands and variability in local performance^9^. Overall, this approach could enhance the accessibility of unstructured clinical information, the comparability to other preexisting studies by using the same clinical rating scale, and the reproducibility by using preexisting instructions streamlining the prompting strategy across studies. By applying LLMs to the extraction of DIP-related information from case reports, this study introduces a novel methodological framework that could be extended to other areas of psychiatry. This underscores the potential of LLMs as powerful tools for systematically analyzing narrative clinical data beyond traditional epidemiological approaches.

## Methods

To identify relevant DIP case reports/series, we performed a literature search using PubMed on DIP in SSD and MOD patients published before May 1st, 2024 (s. supplement). Clinical rater (AG) manually extracted demographic and structured clinical information (i.e., 18 UPDRS motor symptoms)) from the case reports under the supervision of medical expert (DH). Due to the lack of detailed information on the severity of all UPDRS motor symptoms, we assigned a score of 1 to DIP signs if the authors mentioned them and 0 if they did not. For this study, we selected GPT-4o (gpt-4o-2024-05-13) to rate the binarized items and extract demographic information. Subsequently, GPT-4o was applied to automate this process and we tested the congruence of manually and automatically extracted data. Difference in frequency was statistically compared with Chi-squared tests. Significance level was set at 0.05. *P*-values were corrected using the Bonferroni-method. GPT was additionally instructed to evaluate its confidence in the extracted information. GPT-4o was chosen because it performed well in different clinical benchmark tests such as medical exam-style question answering^10^. Additionally, chain-of-thought (CoT) prompting was utilized by adding “think step-by-step” to the prompt^11^. A prompt was generated for each item and demographic information, and each prompt was used in a separate API call. For less random and more reproducible outputs, a temperature of 0.2 and top_*p* value of 0.1 was chosen. For more information on the model settings and prompting strategy see the supplementary material. To evaluate the agreement of the clinical rater and GPT-4o we evaluated the distribution of symptoms, the accuracy of the GPT-generated output, and if simple correlations and group differences remained significant in the GPT-generated output.

## Results

**Fig. 1:**
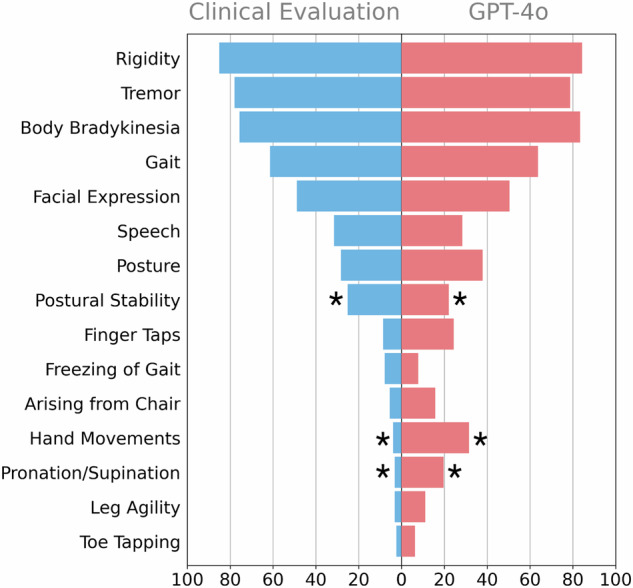
Comparison of the prevalence of UPDRS motor signs as identified by manual curation versus GPT. Asterisk (*) indicate a significant difference between both evaluations.

**Fig. 2:**
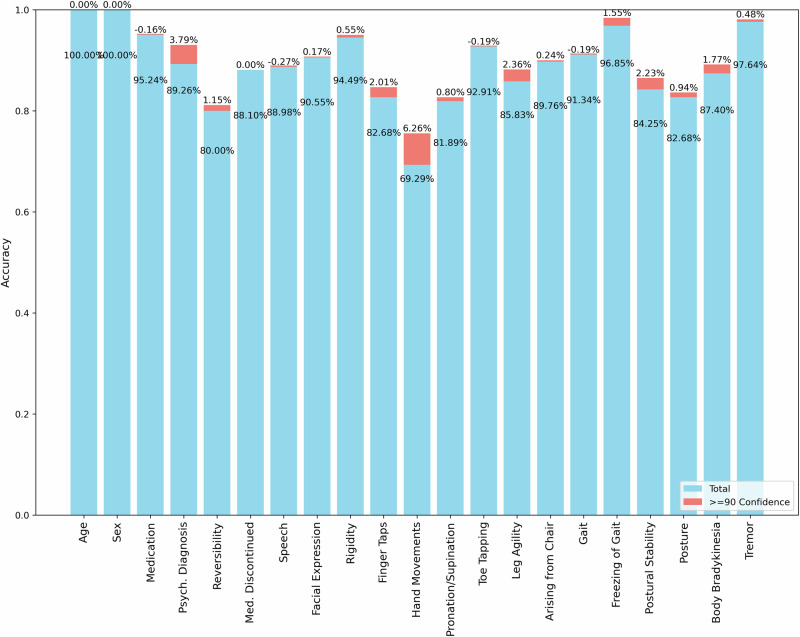
Accuracy of GPT-based reconstruction compared to manual curation. The blue bars represent accuracy before filtering by confidence level, while the red bars indicate the percentage gain in accuracy after applying confidence-based filtering.

We identified 94 DIP case reports (supplement for search strategy), related to 132 SSD and MOD patients from which five cases were dropped, due to insufficient information in the case reports resulting in 127 cases. Sex was balanced with 54% female and 46% male patients. More than half of the patients were at least 50 years old (57.5%), and the biggest group was over 65 years old (36.2%) (supplementary Fig. 1). DIP patients who received antipsychotics were more evenly distributed across age (supplementary Fig. 2), and DIP patients who were generally older, 90,6% were over the age of 40 (supplementary Fig. 3). For over half of the patients (62.6%), antipsychotics were the reason for parkinsonism symptoms, of which 34.6% received second-generation antipsychotics. The largest antidepressant group was serotonin or norepinephrine modulator with 19.7% (supplementary Fig. 4). We found a similar distribution of DIP signs between manually and GPT-4o extracted information (Fig. 1), resulting in the same distribution of demographic variables sex and age. For both approaches, rigidity, tremor, and bradykinesia were the most common symptoms. During the curation of the manually extracted data, the experts found it difficult to distinguish between the four tremor items due to the lack of specificity in the case reports. Therefore, these four items (“Postural tremor of hands”, “kinetic tremor of hands”, “rest tremor amplitude”, “constancy of rest tremor”) were summarized. This resulted in an overall agreement between clinical rater and LLM across all demographic and clinical information of 89.05%. The agreement of only UPDRS items was 87.77%. When filtering for GPT-created confidence levels, namely for 90%, 80%, 50%, and 10% confidence, we observed an increase in agreement when only considering answers GPT evaluated as 90% confident. This resulted in an overall agreement of 90.12% and 89.01% (Fig. 2). An improvement in agreement was not observed when filtering for lower confidence levels. One of the highest agreements was observed in the summarized tremor item with 98.11%. However, when evaluating the four different tremor items separately, and creating an evaluation with a lack of information, the agreement between GPT and experts was comparatively low, namely 60.18%, 81.08%, 32.04%, and 34.69%. The agreement of the evaluation on the individual case report level showed a range of 53.3% to 100%. This agreement did not correlate with the number of signs in the case report, however, it correlated negatively with the number of patients described in the case report (Spearman *r* = −0.25, *p* = 0.01). Notably, 32/94 reports were 100% rated in the same way, and 66/94 were at least 90% rated the same. When looking at significant associations in the data we found in the manually curated dataset a significant difference in facial expression between medication groups (*p* = 0.01) and an association between age and the sum of UPDRS signs (*p* = 0.04). The group difference in facial expression remained significant with a *p* value of 0.02, when using the general tremor item to create the sum of UPDRS symptoms the association with age was not significant anymore. When using all four tremor items the age association was also insignificant (*p* = 0.055).

## Discussion

This study is the first to demonstrate that LLMs can systematically and efficiently analyze extensive collections of DIP case reports/series in a user-friendly manner. This LLM-based method enabled the direct extraction of demographic and clinical (e.g., UPDRS items) data from case reports into a structured format, utilizing decision confidence levels, and pre-existing instructions. The quantitive analysis of case reports seems promising, showing the same most common motor parkinsonism symptoms in the literature, namely rigidity, tremor, and bradykinesia^12,13^. We observed a similar distribution in demographic variables and a high accuracy in identifying DIP symptoms, underscoring the effectiveness of automatic curation and the capability of LLMs as accurate zero-shot reasoners. However, human validation remains crucial, particularly for tremor-related items, due to variations in severity levels. Conducting a preliminary sample assessment, clearly defining tremor severity categories, and thoroughly evaluating the extracted details from the unstructured text would likely improve both accuracy and reliability.

The use of LLMs must adhere to strict legal and ethical guidelines, particularly to prevent the inadvertent disclosure of sensitive data^14–16^. A key ethical consideration in our study is that patients described in the analyzed case reports did not explicitly consent to AI-based analysis. However, in our approach, the risk of personal data identification remains minimal, as the analyzed information is already publicly available and further anonymized through LLM processing. Nevertheless, we acknowledge broader ethical concerns associated with LLM-driven analysis of case reports and clinical records, particularly the risk of re-identification when such data are combined with external sources, including health insurance records or social media data.

### Limitations

This study has several limitations that warrant consideration. First, we did not systematically evaluate smaller language models or rigorously explore different parameter settings (e.g., temperature, top_p) to identify the optimal configuration; however, our 90% agreement rate suggests sufficient performance for our purposes. Second, although we assume that user satisfaction correlates with reduced manual labor in case report searches, we did not formally assess usability or user experience—future investigations should address this. Third, our review was primarily narrative rather than strictly systematic, even though we focused on case reports using a systematic search in PubMed (restricted to English and German). Consequently, relevant data in other databases or languages may have been overlooked. Fourth, we relied on single ratings of each report without multiple raters or repeated assessments; incorporating inter-rater reliability tests and consistency checks in future work would strengthen confidence in our findings. Fifth, unreported symptoms by the original physicians may lead to missed associations, and we assumed that absence of documentation equated to absence of the symptom. In addition, literature search was performed manually in PubMed, though future research might automate and broaden this process using more advanced or evolving LLMs. Finally, LLMs continue to improve, exemplified by the more sophisticated reasoning of recently released models such as GPT-o1. These ongoing developments hold promise for refining automated data extraction, offering enhanced diagnostic insights, and potentially transforming clinical practice for underrepresented conditions. Despite these constraints, our findings highlight the promise of LLM-based approaches and provide a foundation for further systematic investigation.

## Conclusion

This proof-of-concept study highlighted the potential of LLMs to augment human expertise in psychiatric research and clinical practice. This work also expanded on the concept of automatic evaluation instructing GPT to evaluate its own confidence. The automated conversion of unstructured medical information into structured form may substantially support personalized medicine, and help refine diagnostic criteria, e.g., with respect to DIP.

## Supplementary information

Supplementary material methods

Supplementary figures

## Data Availability

The datasets used and/or analyzed during the current study available from the corresponding author on reasonable request.

## Code availability

The underlying code for this study is not publicly available but may be made available to qualified researchers on reasonable request from the corresponding author.
