# Supplementary material for: Validating large language models against manual information extraction from case reports of drug-induced parkinsonism in patients with schizophrenia spectrum and mood disorders: a proof of concept study
Source: Schizophrenia (Heidelb). 2025 Mar 20;11(1):47. doi: 10.1038/s41537-025-00601-5 (PMC11926372; doi:10.1038/s41537-025-00601-5)
Supplement: Supplementary file 1 — Supplementary material methods [file 41537_2025_601_MOESM1_ESM.docx]

**Supplementary material**

**Methods**

**Systematic literature search**

For the purpose of this narrative review, a search was performed in PubMed for case reports and case series published before Mai 1^st^, 2024. The following terms were used: “case reports” (separated by OR) in combination with the terms “parkinsonism”, “drug-exacerbated parkinsonism”, "drug-induced parkinsonism" and “medication-induced parkinsonism” (separated by OR). We selected articles that: (i) were published in a peer-reviewed journal in English or German and (ii) included patients with schizophrenia spectrum (SSD) or mood disorders (MOD). Case reports/series describing patients with severe white matter hyperintensities, multiple lacunes in the basal ganglia, tumor, or hydrocephalus on brain MRI; and other major neurologic (epilepsy, stroke, encephalitis, or dementia) or metabolic illness were excluded from this review. Of note, case reports/series on isolated sensorimotor abnormalities such as tremor, myoclonus, asterixis or ataxia were not included in this review, and case reports/series that did not focus on the overall clinical picture of parkinsonism were excluded as well, because these may have partly distinct underlying mechanisms. The manual analysis and data extraction were conducted by A.G., under the supervision of D.H., an experienced psychiatrist and researcher specializing in genuine and drug-induced sensorimotor abnormalities in psychiatric disorders. During the manual analysis of the identified case reports, the four tremor items from the **Unified Parkinson's Disease Rating Scale (UPDRS)** were consolidated into a single item. A score of 1 was assigned if any one of the four tremor items was mentioned in the case report, due to the difficulty in distinguishing between them.

**Model Settings**

The model was configured with a temperature of 0.2, a top_p value of 0.1, and a maximum token output of 4096. The temperature parameter regulates the model's "randomness", where higher values result in more diverse outputs. The top_p parameter employs a sampling technique that selects the next word from the smallest possible set whose cumulative probability meets or exceeds the specified probability value (p). The max_tokens parameter defines the upper limit of generated output tokens. The model's system role was designated as "You are a helpful assistant." For a comprehensive description of these parameters, we refer readers to the OpenAI official documentation. Different prompts, maintaining a consistent structure, were used for each table entry.

The structure of the prompts were as follows:

1. We started with “Think step by step,...”
2. “could you evaluate” followed by the demographic information, e.g., “age and sex of the patients” or “given the Parkinson's sign described in the following:”
3. Optionally, the defined Parkinsonism sign defined by the UPDRS. With the addition of “Please do not rate a symptom as 1 unless it is explicitly mentioned in the report.”
4. “Please provide an individual assessment for each patient, with a separate evaluation of” either “symptom present” or demographic information followed by “and the corresponding level of certainty.”
5. “Additionally, assess your level of certainty regarding your answer on a scale from 0 to 100, where 0 signifies complete uncertainty and 100 signifies complete certainty.\n”
6. “Please provide an individual assessment for each patient, with a separate evaluation of age and sex, and the corresponding level of certainty. \n”
7. “Here is the case report:\n”
8. This was then followed by a processed case report. The processing included removal of any logos and figures, and removing the references. The case report had therefore a raw text format.

Instructions included: (I) a separate prompt for each of the 18 different MDS-UPDRS motor-items, a prompt age and sex (II) a prompt for reversibility of Parkinsonism signs defined with complete remission, partial remission/lost to follow-up, or signs persisted (III) if the psychopharmacological drug was discontinued, reduced, and if additional medication was described. However, only the answer to “discontinued” was further evaluated (IV) diagnosis of the patient according to the ICD-10 of either schizophrenia spectrum disorder (F2X), mood disorder (F3X), or not defined.

**Temperature and top_p selection**

By reducing both the temperature and top_p parameters, we observed a decline in the “creative” variability of GPT-4’s outputs. This decrease in linguistic divergence produced more consistent text, which in turn facilitated the generation of CSV files. Below is an example output generated under these lower-randomness conditions:

### Summary

- **Age:** 32 years old

- **Certainty Level:** 100

- **Sex:** Male

- **Certainty Level:** 100

Additionally, we initially tested higher randomness values in combination with a majority-voting approach—generating the same prompt multiple times and selecting the most frequent response. However, extracting information from these outputs proved challenging, prompting us to return to lower randomness settings. Below are two example outputs of the same prompt produced under these higher randomness conditions, showing the inconsistency when using the same prompt:

First Output:

### Final Assessment:

Age: 32 years old (Certainty: 100%)

Sex: Male (Certainty: 100%)

Second Output:

Age Evaluation:

The report explicitly states that the patient is a 32-year-old.

Certainty level: 100% (Age is clearly mentioned in the case presentation).

Sex Evaluation:

The report consistently refers to the patient as a male (e.g., "young male veteran").

Certainty level: 100% (Sex is explicitly stated in multiple instances).

Thus, the patient is a 32-year-old male, with 100% certainty for both assessments.

**Ethical approval**

This study is based solely on previously published research and does not involve any new studies with human participants or animals conducted by the authors. Consequently, ethical approval was not necessary for this study.

**Informed consent**

This study relies exclusively on previously published research and does not involve any new studies with human participants conducted by the authors. As such, informed consent was not required.

**UPDRS-Sign Example Prompt**

Think step by step, given the Parkinson's sign described in the following:\n3.1 SPEECH\nInstructions to examiner: Listen to the patients free-flowing speech and engage in conversation if \nnecessary. Suggested topics: ask about the patients work, hobbies, exercise, or how he got to the \ndoctors office. Evaluate volume, modulation (prosody), and clarity, including slurring, palilalia (repetition \nof syllables), and tachyphemia (rapid speech, running syllables together).\n0: Normal: No speech problems.\n1: Slight: Loss of modulation, diction, or volume, but still all words easy to understand.\n2: Mild: Loss of modulation, diction, or volume, with a few words unclear, but the overall \nsentences easy to follow.\n3: Moderate: Speech is difficult to understand to the point that some, but not most, sentences are \npoorly understood.\n4: Severe: Most speech is difficult to understand or unintelligible.\nPlease indicate if this symptom was present by answering '1' for present and '0' for not present. Additionally, assess your level of certainty regarding your answer on a scale from 0 to 100, where 0 signifies complete uncertainty and 100 signifies complete certainty.\nPlease do not rate a symptom as 1 unless it is explicitly mentioned in the report.\nPlease provide an individual assessment for each patient, with a separate evaluation of symptom presence and the corresponding level of certainty.\n\nHere is the case report:

**Demographic Example Prompt**

Think step by step, could you evaluate age and sex of the patients:\nAdditionally, assess your level of certainty regarding your answer on a scale from 0 to 100, where 0 signifies complete uncertainty and 100 signifies complete certainty.\nPlease provide an individual assessment for each patient, with a separate evaluation of age and sex, and the corresponding level of certainty.\n\nHere is the case report:\n
