# Supplementary material for: Validating large language models against manual information extraction from case reports of drug-induced parkinsonism in patients with schizophrenia spectrum and mood disorders: a proof of concept study
Source: Schizophrenia (Heidelb). 2025 Mar 20;11(1):47. doi: 10.1038/s41537-025-00601-5 (PMC11926372; doi:10.1038/s41537-025-00601-5)
Supplement: Supplementary file 2 — Supplementary figures [file 41537_2025_601_MOESM2_ESM.docx]

**Supplementary figures.**


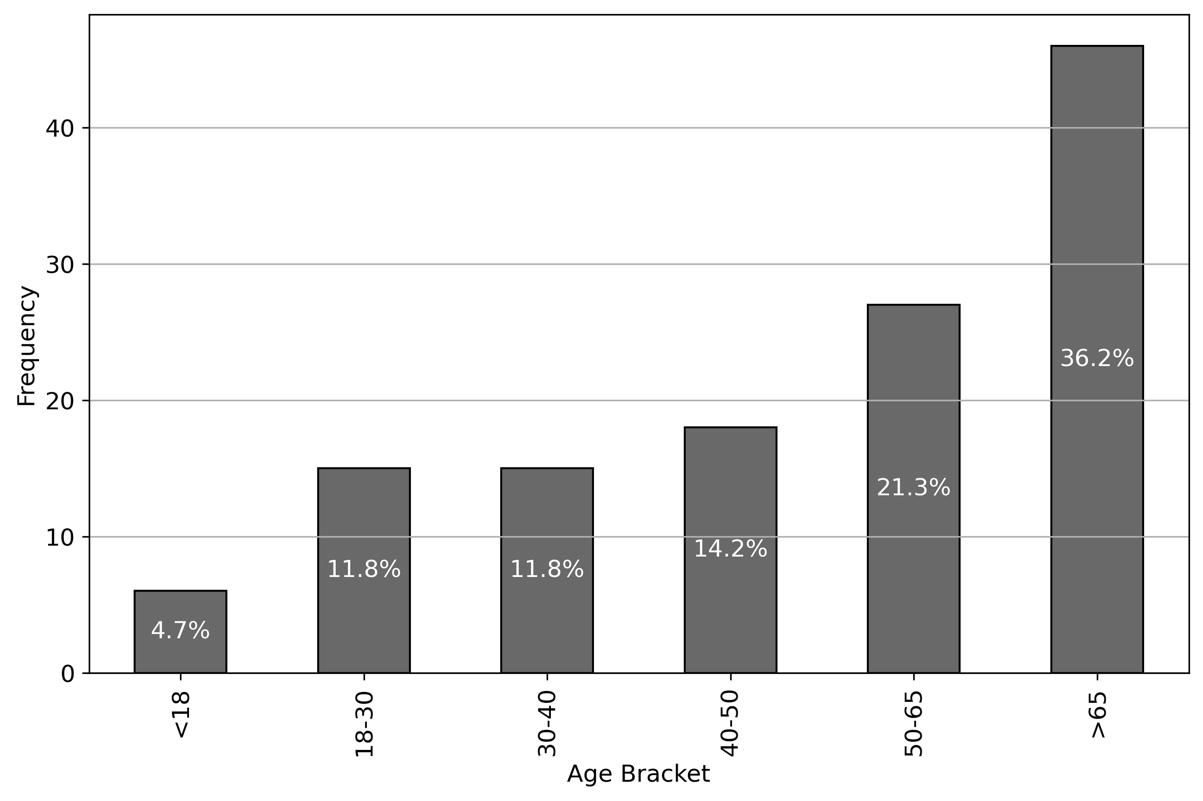


**Supplementary Figure 1.** Age distribution of the total cohort of drug-induced parkinsonism patients that received mood stabilizer, antidepressants, or antipsychotics.


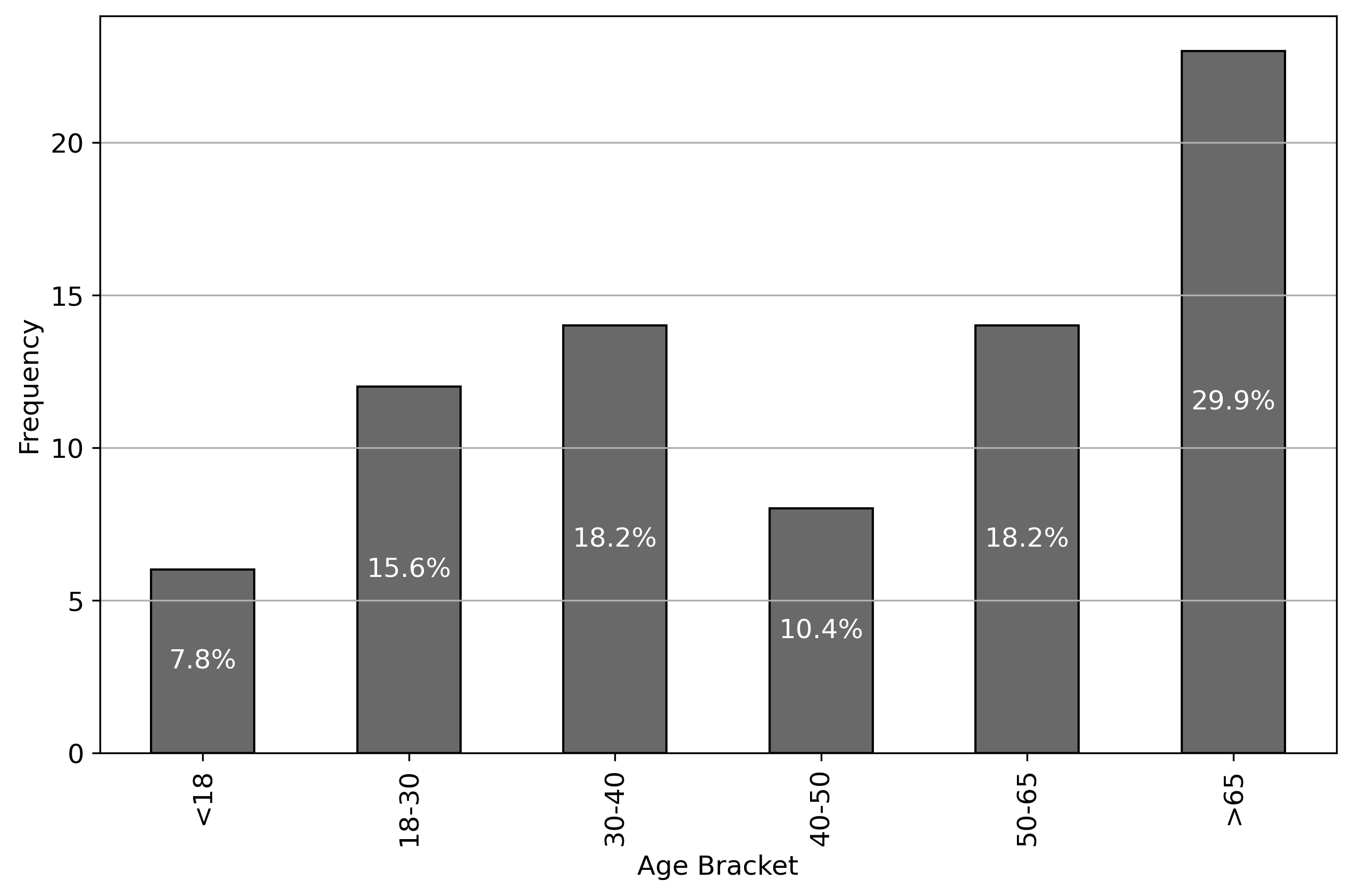


**Supplementary Figure 2.** Age distribution of the total cohort of drug-induced parkinsonism patients that received antipsychotics.


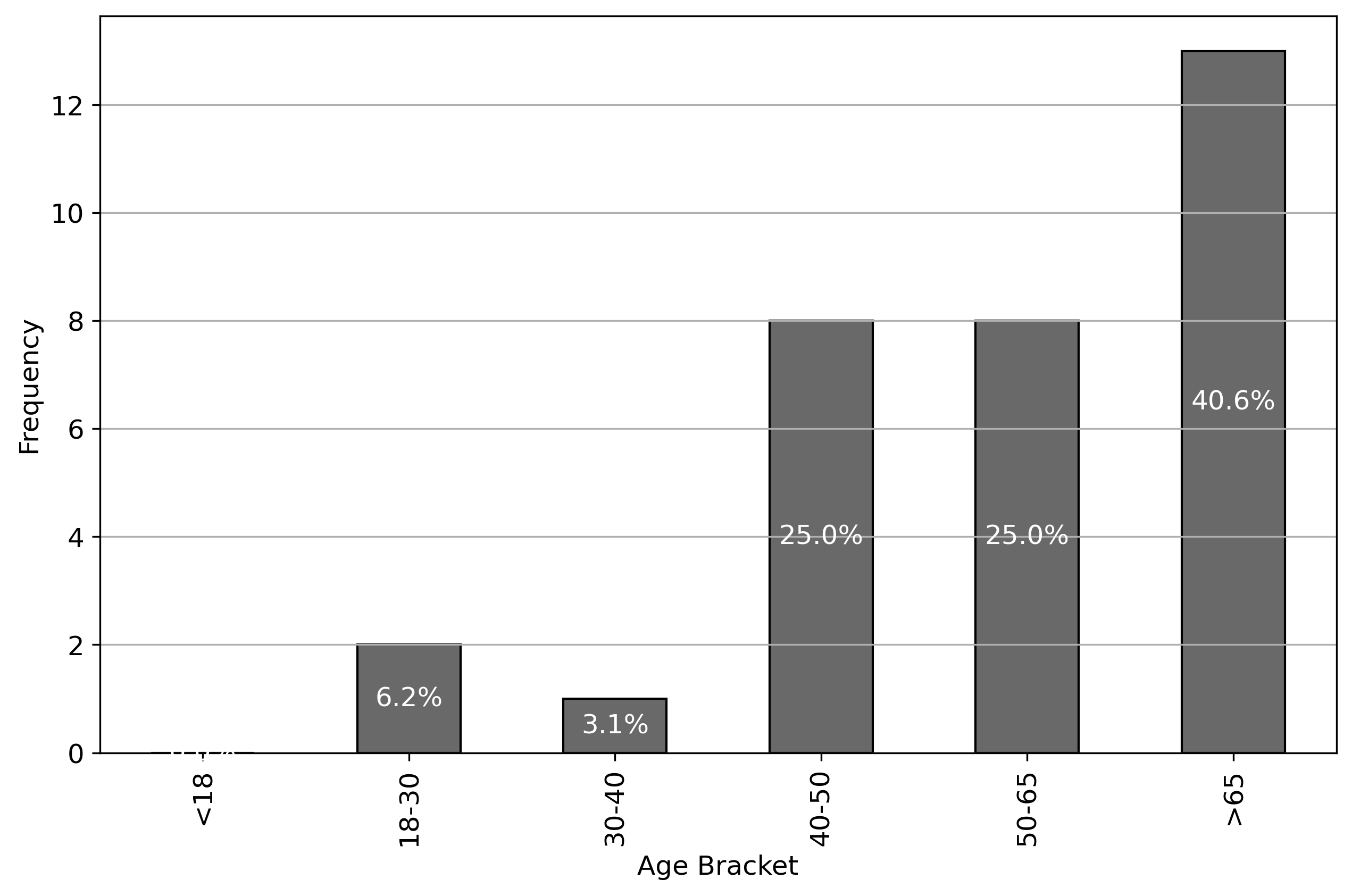


**Supplementary Figure 3.** Age distribution of the total cohort of drug-induced parkinsonism patients that received antidepressants.


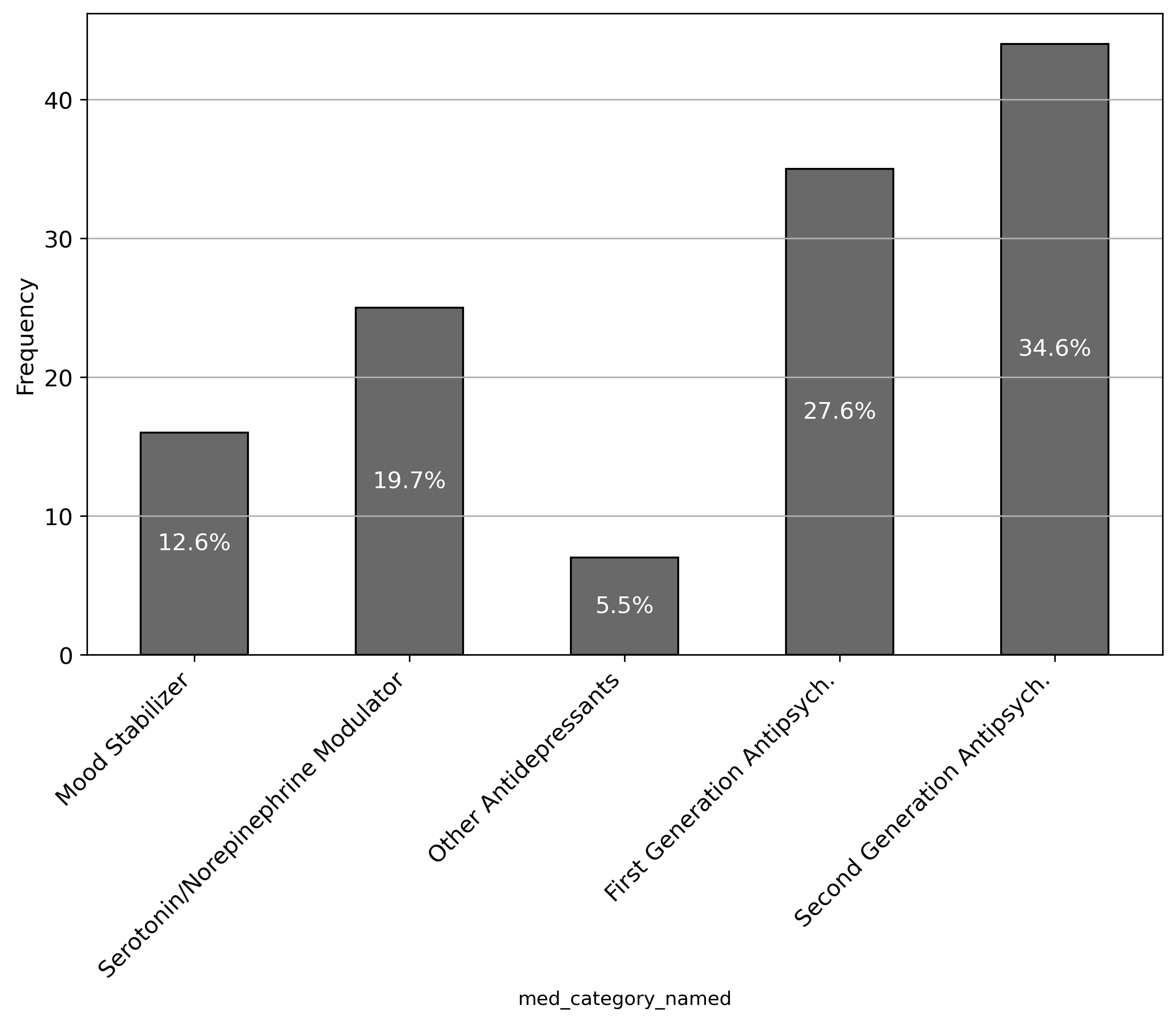


**Supplementary Figure 4.** Distribution of the medication categories that induced parkinsonism signs.
